# Tailored total lymphoid irradiation in heart transplant patients: 10-years experience of one center

**DOI:** 10.1186/1748-717X-5-3

**Published:** 2010-01-16

**Authors:** Pirus Ghadjar, Daniela Joos, Michele Martinelli, Roger Hullin, Marcel Zwahlen, Kristina Lössl, Thierry Carrel, Daniel M Aebersold, Paul Mohacsi

**Affiliations:** 1 Department of Radiation Oncology, University Hospital Bern, Freiburgstrasse, 3010 Bern, Switzerland; 2 Department of Cardiovascular Surgery, Swiss Cardiovascular Center, University Hospital Bern, Freiburgstrasse, 3010 Bern, Switzerland; 3 Institute of Social and Preventive Medicine, University of Bern, Switzerland, Finkenhubelweg 11, 3012 Bern, Switzerland

## Abstract

**Background:**

To assess safety and efficacy of tailored total lymphoid irradiation (tTLI) in cardiac transplant patients.

**Methods:**

A total of seven patients, of which five had recalcitrant cellular cardiac allograft rejection (RCCAR), confirmed by endomyocardial biopsies, and two had side effects of immunosuppressive drug therapy, were all treated with tTLI. tTLI was defined by the adjustment of both the fraction interval and the final irradiation dosage both being dependent on the patients general condition, irradiation-dependent response, and the white blood and platelet counts. A mean dose of 6.4 Gy (range, 1.6 - 8.8 Gy) was given. Median follow-up was 7 years (range, 1.8 - 12.2 years).

**Results:**

tTLI was well tolerated. Two patients experienced a severe infection during tTLI (pneumocystis jirovecii pneumonia, urosepsis and generalized herpes zoster) and one patient developed a lymphoproliferative disorder after tTLI. The rate of rejection episodes before tTLI was 0.43 episodes/patient/month and decreased to 0.02 episodes/patient/month after tTLI (P < .001). At the end of the observation time, all patients except one were alive.

**Conclusions:**

tTLI is a useful treatment strategy for the management of RCCAR and in patients with significant side effects of immunosuppressive drug therapy. In this series tTLI demonstrated significantly decreased rejection rates without causing relevant treatment-related toxicity.

## Background

Recalcitrant cellular cardiac allograft rejection (RCCAR) is one of the remaining unsolved problems after heart transplantation (HTx). In case of RCCAR, aggressive up-regulation or adaptation of immunosupression is required, taking additional toxicity into account [1,2], however, without being sufficient enough to control the rejection process. Total lymphoid irradiation (TLI) for the treatment of RCCAR has been established for over two decades with several published reports demonstrating safety and efficacy of this technique [2-18]. However, little is known about the long term effects after TLI [12,17]. This study reports on the long-term outcome of seven patients after HTx who received tailored TLI (tTLI) with a median follow-up of 7 years.

## Methods

### Patients

Five patients with RCCAR (defined as a rejection grade of IIIA in at least three consecutive endomyocardial biopsies (EMBs) as defined by the International Society for Heart and Lung Transplantation (ISHLT) criteria [19]) and two patients with severe side effects to immunosuppressive drug treatment were treated by tTLI between February 1996 and January 2006. All patients who underwent HTx at the Swiss Cardiovascular Center, Inselspital, Bern University Hospital received maintenance immunosuppressive therapy with cyclosporine (n = 6), tacrolimus (n = 3), sirolimus (n = 4), prednisone (n = 7), mycophenolic mofetil (n = 5) or azathioprine (n = 4). One patient received a murine antihuman mature T-cell monoclonal antibody (OKT3) for a total of 9 days, 3 weeks prior to tTLI, because of ongoing rejection. All patients received as induction a polyclonal therapy with rATG (Fresenius) during the first 5 days after HTx. Patients were analyzed retrospectively. This study was performed in accordance with the standards of the local ethics committee and with the Helsinki Declaration.

### Radiation therapy

The TLI was performed tailored as previously described [9]. Briefly, patients were treated by three separate fields to encompass all major lymph node bearing areas. A supradiaphragmatic mantle field, a periaortic and splenic field and a pelvic field with inferior extension were used in two patients. The remaining five patients received a supradiaphragmatic mantle field and a inverted Y-field. The non-lymphoid tissue was appropriately shielded in all fields. All fields were treated concurrently on a linear accelerator by an anterior/posterior opposed technique with 6 and 15 MV photons. One patient was treated with a Cobalt 60 source. The prescribed dose was 8 Gy delivered in individual twice weekly 0,8 Gy fractions (calculated midplane dose at central ray). tTLI was performed on a Monday/Thursday or Tuesday/Friday schedule. A control EMB was performed after 2 - 4 fractions. The final decision regarding the dosing was based on the clinical state (e.g. infection), the irradiation-dependent response (efficacy) as well as on the white blood count (WBC) and/or platelet count (Plts) on the day of or one day before planned tTLI. In general tTLI was withheld for a total WBC less than 2.7 thousand/mm^3 ^(or total granulocyte count less than 1.5 thousand/mm^3^) or Plts less than 125 thousand/mm^3 ^or if Plts were rapidly decreasing.

### Statistical analysis

Descriptives include absolute and relative frequencies for categorical variables and the mean and standard deviation for quantitative variables. The primary objective of this study was to analyze safety and efficacy of tTLI. Secondary objective was to assess long-term survival and toxicity. A severe infection was defined as requiring hospitalisation and/or intravenous antibiotics.

All available WBC, Plts and haemoglobin (Hb) data were collected and analyzed from the time of HTx until 1 year after tTLI. All post-transplant rejection and infection episodes were recorded beginning right after HTx and ending at the end of the follow-up period for each patient. The analysis of tTLI rejection rates was based on calculating the proportion of positive biopsies before and after tTLI and done using an extension of generalized linear models for binomially distributed data using the identity link. Statistical significance was considered on a two-sided level of α = 0.05.

The calculations were performed with the Statistical Analysis Systems (SAS) package (SAS Institute, Cary, NC, USA, version 9.1).

## Results

### Patients

Patient characteristics are summarized in Table 1. Four patients were male, three were female. Median age at HTx was 47 years (range, 19 - 62 years). After HTx and before tTLI patients had undergone a median of 14 EMBs (range, 7 - 37 EMBs) which detected a median of 5 rejections graded IIIA (range, 3 - 12 rejections). Median time from HTx to TLI was 8.8 months (range, 1.6 - 36 months). Prior to tTLI five patients had a history of at least one severe infection. Three patients had cytomegalovirus infection. One patient had pneumocystis jirovecii pneumonia and one year later generalized herpes zoster. Another patient had gram-negative sepsis. All infections resolved after therapy with appropriate antibiotics. The mean dose was 6.4 Gy (range, 1.6 - 8.8 Gy) with the mean total tTLI duration being 44.6 days (range, 3 - 67 days). During tTLI a median of 4 EMBs were performed (range, 1 - 6 EMBs). In one patient tTLI was discontinued because of leukocytopenia and thrombocytopenia and continued after 1.5 months. In one patient tTLI was discontinued as EMBs revealed no evidence of rejection. However one month later tTLI was again required for reoccurrence of rejection (Table 1). Regarding the immunosuppressive drug therapy during tTLI, cyclosporine was continued in 4/6 patients, tacrolimus in 2/3 patients, sirolimus in 2/4 patients, mycophenolic mofetil in 3/5 patients and azathioprine in 3/4 patients.

**Table 1:** Patient characteristics

Patient	Gender	Age at HTx (years)	Dx at HTx	Interval between HTx and tTLI (months)	Duration of tTLI (days)	Number of tTLI courses	tTLI dose (Gy)	Follow-up (years)
1	Male	34	DCMP	10.7	67	2	4 + 4.8*	7.0
2	Female	57	HOCMP	36	48	1	6.4	5.2
3	Female	43	Myocarditis	2.7	3	1	1.6	1.8
4	Male	19	CHD	33.6	43	1	8	1.9
5	Male	47	DCMP	1.6	27	1	4.8	11.7
6	Female	62	CVD	2.4	57	2	1.6 + 6.4^+^	12.2
7	Male	55	CAD	8.8	67	1	8	10.3

Abbreviations: HTx = heart transplantation; Dx at HTx = diagnosis leading to heart transplantation; DCMP = dilatative cardiomyopathia; HOCMP = hypertrophic obstructive cardiomyopathia; CHD = congenital heart disease; CVD = cardiac valvular disease; CAD = coronary artery disease; tTLI = tailored total lymphoid irradiation. * Second tTLI was initiated 1.5 months after the first one or 12.5 months after HTx. ^+^Second tTLI was initiated 1 month after the first one or 3.5 months after HTx.

### Evaluation of Safety

**Figure 1:**
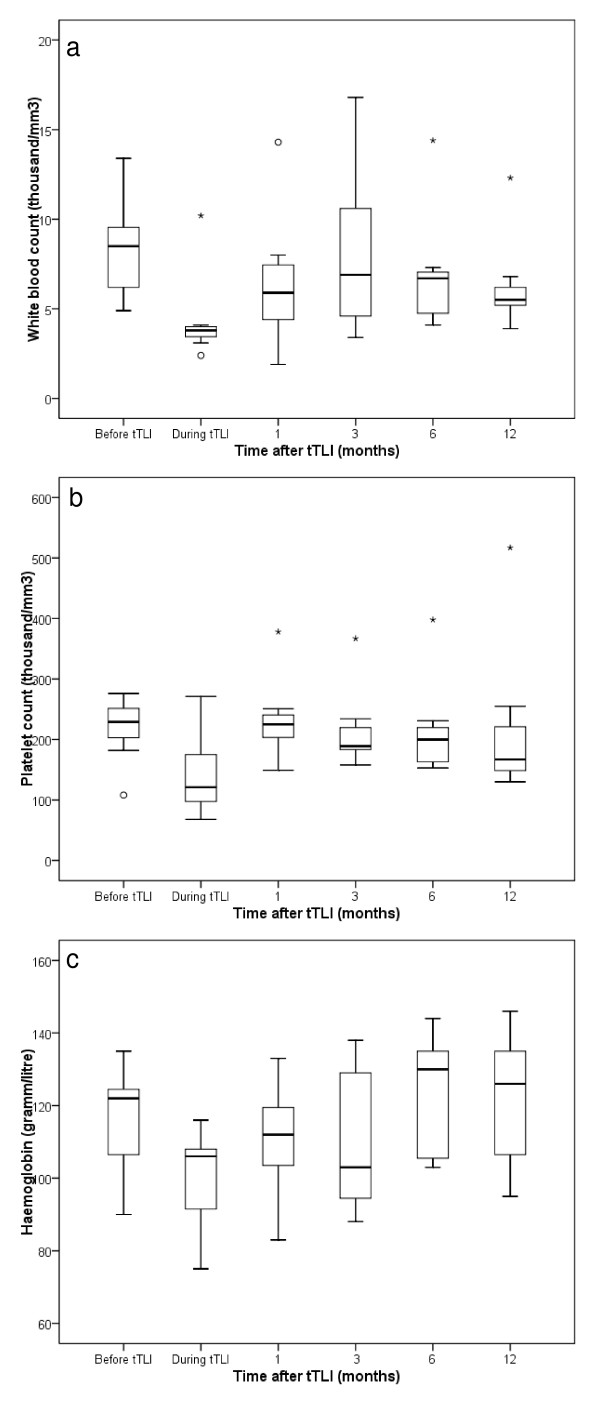
**White blood count (a), platelet count (b) and haemoglobin values (c) before, during (lowest value observed during tTLI) and 1, 3, 6, and 12 months after tTLI**. Boxes represent 25% and 75% percentiles. Circles indicate outlier, asterisks indicate extreme values.

General side effects during tTLI were mild and limited to fatigue and epigastric pain. Transient bone marrow suppression occurred in all patients. Three patients experienced transient leukocytopenia of < 3.5 thousand/mm^3 ^(range, 1.4 - 3.1 thousand/mm^3^) and five patients experienced transient thrombocytopenia of < 140 thousand/mm^3 ^(range, 68 - 130 thousand/mm^3^). The median time to the nadir of WBC, Plts and Hb was 1.5 months, 0.8 months and 0.5 months with the mean nadir values being 4.1 thousand/mm^3 ^(range, 2.1 - 10.2 thousand/mm^3^), 123.4 thousand/mm^3 ^(range, 68 - 241 thousand/mm^3^) and 95.9 gramm/litre (range, 75 - 116, gram/litre), respectively. Virtually, all bone marrow functions recovered within the three months post-tTLI (Figure 1). During tTLI only two patients suffered from severe infection. One patient with leukocytopenia experienced pneumocystis jirovecii pneumonia and urosepsis and another patient suffered from generalized herpes zoster. All infections were successfully treated. Further, there were no deaths observed during or immediately following the tTLI. However, one patient died almost 5 years after HTx and 1.9 years after completion of tTLI because of graft coronary artery disease, all other patients were alive at the end of follow-up. One patient developed post-transplant lymphoproliferative disorder 5 months after HTx and 2.4 months after completion of tTLI. This patient had undergone OKT3 treatment prior to the tTLI.

### Evaluation of Efficacy

The rejection episodes before, during and after tTLI are summarized in Table 2.

**Table 2:** Number of rejection episodes before, during and after tTLI

Patient	Rejection episodes before tTLI*	Rejection episodes during tTLI*	Rejection episodes after tTLI*	Time to first subsequent rejection episode (months)	Severe Infections during tTLI	Current status
1	7	1	1	31	HZ	alive
2	5	2	1	25	none	alive
3	5	0	1	5	none	alive
4	12	0	0	n.a.	none	dead#
5	3	0	2	77	US, PJP	alive+
6	4	1	6	29	none	alive
7	5	0	1	31	none	alive

Abbreviations: tTLI = tailored total lymphoid irradiation; n.a. = not applicable; HZ = generalized herpes zoster; US = urosepsis; PJP = pneumocystis jirovecii pneumonia; * Rejection episodes were defined as a rejection of ≥ IIIA according to the International Society for Heart and Lung Transplantation (ISHLT) criteria; # patient died because of graft coronary artery disease; + patient experienced a post-transplant lymphoproliferative disorder.

The rate of rejection episodes before tTLI was 0.43 episodes/patient/month (95% confidence interval [CI]: 0.31 - 0.58) and decreased to 0.02 episodes/patient/month after tTLI (95% CI: 0.01 - 0.034; P < .001). During the 4 months after tTLI none of the seven patients had further rejection episodes. In the long-term perspective, treatment with tTLI resulted in a decrease of rejection episodes by 28.6% (95% CI: 20.1 - 37.1% decrease). The median time from tTLI to the first subsequent rejection episode was 2.4 years. On average, patients have remained free from acute rejection for 4.1 years (range, 1.4 - 7.8 years).

## Discussion

This study describes long-term safety and efficacy of tTLI in patients who have experienced RCCAR or toxicity of immunosuppressive drug therapy after HTx. In accordance with the literature, describing mainly a non-tailored approach, tTLI was shown to effectively reduce the rejection rate without major treatment related toxicity or infections after a median follow-up of 7 years. Potential long-term risks of tTLI include radiation-induced cardiomyopathy and graft coronary artery disease [17]. The patient who had died from graft coronary artery disease in our series had undergone AB0 mismatched HTx, as previously described [20]. In our series one patient developed a post-transplant lymphoproliferative disorder after he had received OKT3 treatment three weeks prior to the tTLI. The post-transplant lymphoproliferative disorder occurred 2.4 months after completion of tTLI which was diagnosed as an Epstein B Virus (EBV) associated extranodal B-cell lymphoproliferative disease located in the left lung. OKT3 treatment has been shown to be associated with an increased incidence of lymphoproliferative diseases [21,22]. There however remains a possibility that the lymphoproliferative disorder was attributable to the increased immunosuppression achieved by tTLI. The actuarial risk for post-transplant lymphoproliferative disorder at 5-years after TLI was described to be 9% [23]. It has been demonstrated by others that mTOR-inhibition (S6 kinase pathway), decreases the apoptotic threshold. This may lead to a hypersensitivity of tissues to radiotherapy in patients treated with an mTOR-inhibitor [24,25]. Since a significant proportion of HTx patients are treated with mTOR inhibitors today, the interaction between mTOR-inhibition and tTLI is an important issue. However, in vitro observations were based on anti-cancer radiation doses, as a consequence the impact of mTOR-inhibition together with the relative low radiation dose used in TLI may not be of clinical importance. In accordance, four of our patients were treated with sirolimus prior to tTLI two of which continued sirolimus during tTLI. In our study there was no difference in WBC or Plts in the patients who received tTLI in combination with sirolimus compared to the patients who received tTLI without sirolimus. Nevertheless, if tTLI is performed in patients on mTOR-inhibitors, careful monitoring is still warranted. The main limitation of this study is that it is a retrospective analysis of a relatively small patient cohort with individually tailored immunosuppressive drug treatment. Our experience has however demonstrated an acceptable safety profile and good efficacy of tTLI which we only instigate in patients with RCCAR already on aggressive immunosuppressive drug treatment or those who cannot tolerate these newer agents (tacrolimus, mycophenolic mofetil and mTOR-inhibitors). We have also demonstrated the feasibility of combined treatment with tTLI and the newer immunosuppressive drugs but recommend careful clinical monitoring.

## Conclusion

Based on our center's experience, tTLI is a useful treatment strategy with acceptable safety and good efficacy for both management of RCCAR and for the treatment of patients with limited tolerance to their immunosuppressive drugs after HTx.

## Competing interests

The authors declare that they have no competing interests.

## Authors' contributions

Each author had participated sufficiently in the work to take public responsibility for appropriate portions of the content. PG, DJ, DMA and PM designed the study. PG, DJ and MZ performed the statistical analysis. PG, DJ, MM, RH, MZ, KL, TC, DMA and PM collected the data and interpreted the data. The manuscript was written by PG and PM, all other authors helped and finally approved the final manuscript.

## References

[B1] Kirklin JK Naftel DC McGiffin DC McVay RF Blackstone EH Karp RB Analysis of morbid events and risk factors for death after cardiac transplantation J Am Coll Cardiol 1988 11 917 924 3281995 10.1016/s0735-1097(98)90045-6

[B2] Lim TS O Driscoll G Freund J Peterson V Hayes H Heywood J Short-course of total lymphoid irradiation for refractory cardiac transplantation rejection J Heart Lung Transplant 2007 26 1249 54 10.1016/j.healun.2007.09.002 18096475

[B3] Frist W Winterland A Gerhardt EB Merrill WH Atkinson JB Eastburn TE Stewart JR Eisert DR Total lymphoid irradiation in heart transplantation: adjunctive treatment for recurrent rejection Ann Thorac Surg 1989 48 863 4 2596925 10.1016/0003-4975(89)90689-9

[B4] Macoviak JA Darrell J Simmons J Low dose total lymphoid irradiation for recalcitrant myocardial rejection J Heart Lung Transplant 1990 9 76

[B5] Hunt SA Strober S Hoppe RT Strinson EB Total lymphoid irradiation for treatment of intractable cardiac allograft rejection J Heart Lung Transplant 1991 10 211 6 2031918

[B6] Salter MM Kirklin JK Bourge RC Naftel DC Total lymphoid irradiation in the treatment of early or recurrent heart rejection J Heart Lung Transplant 1992 11 902 12 1420238

[B7] Evans MA Schomberg PJ Rodeheffer RJ Katzmann JA Schnell WA Jr Tazelaar HD McGregor CG Edwards BS Total lymphoid irradiation: a novel and successful therapy for resistant cardiac allograft rejection Mayo Clin Poc 1992 67 785 90 10.1016/s0025-6196(12)60804-0 1434918

[B8] Kirklin JK George JF McGiffin DC Naftel DC Salter MM Bourge RC Total lymphoid irradiation: is there a role in pediatric heart transplantation? J Heart Lung Transplant 1993 12 S293 300 8312348

[B9] Salter SP Salter MM Kirklin JK Bourge RC Naftel DC Total lymphoid irradiation in the treatment of early or recurrent heart rejection Int J Radiat Oncol Biol Phys 1995 33 83 8 7642435 10.1016/0360-3016(95)00135-L

[B10] Keogh A Morgan G MacDonald P Spratt P Mundy J McCosker C Clinical heart transplantation. Total lymphoid irradiation for resistant rejection after heart transplantation: only moderate success medium-term J Heart Lung Tranplant 1996 15 231 3 8777203

[B11] Madden BP Backhouse L McClosky D Reynolds L Tait D Murday A Total lymphoid irradiation as rescue therapy after heart transplantation J Heart Lung Transplant 1996 15 234 8 8777204

[B12] Wolden SL Tate DL Hunt SA Strober S Hoppe R Long term results of total lymphoid irradiation in the treatment of cardiac allograft rejection Int J Radiat Oncol Biol Phys 1997 39 953 60 9392531 10.1016/s0360-3016(97)00504-x

[B13] Ross HJ Gullestad L Pak J Slauson S Valantine HA Hunt SA Methotrexate or total lymphoid radiation for treatment of persistent or recurrent allograft cellular rejection: a comparative study J Heart Lung Transplant 1997 16 179 89 9059929

[B14] Trachiotis GD Johnston TS Vega JD Crocker IR Chesnut N Lutz JF Smith AL Kanter KR Single field total lymphoid irradiation in the treatment of refractory rejection after heart transplantation J Heart Lung Transplant 1998 17 1045 8 9855442

[B15] Madden BP Barros J Backhouse L Stamenkovic S Tait D Murday A Intermediate term results of total lymphoid irradiation for the treatment of non-specific graft dysfunction after heart transplantation Eur J Cardiothorac Surg 1999 15 663 6 10.1016/S1010-7940(99)00042-1 10386414

[B16] Keogh AM Arnold RH MacDonald PS Hawkins RC Morgan GW Spratt PM A randomized trial of tacrolimus versus total lymphoid irradiation for the control of repetitive rejection after cardiac transplantation J Heart Lung Transplant 2001 20 1331 4 10.1016/S1053-2498(01)00329-1 11744418

[B17] Chin C Hunt S Robbins R Hoppe R Reitz B Bernstein D Long term follow-up after total lymphoid irradiation in pediatric heart transplant recipients J Heart Lung Transplant 2002 21 667 73 10.1016/S1053-2498(01)00772-0 12057700

[B18] Asano M Gundry SR Razzouk AJ del Rio MJ Thomas M Chinnock RE Bailey LL Total lymphoid irradiation for refractory rejection in pediatric heart transplantation Ann Thorac Surg 2002 74 1979 85 10.1016/S0003-4975(02)04065-1 12643383

[B19] Billingham ME Cary NR Hammond ME Kemnitz J Marboe C McCallister HA Snovar DC Winters GL Zerbe A A working formulation for the standarization of nomenclature in the diagnosis of heart and lung rejection: Heart Rejection Study Group. The International Society for Heart Transplantation J Heart Lung Transplant 1990 9 587 93 2277293

[B20] Koestner SC Kappeler A Schaffner T Carrel TP Nydegger UE Mohacsi P Histo-blood group type change of the graft from B to 0 after AB0 mismatched heart transplantation Lancet 2004 363 1523 25 10.1016/S0140-6736(04)16179-5 15135601

[B21] Swinnen LJ Costanzo-Nordin MR Fisher SG O'Sullivan EJ Johnson MR Heroux AL Dizikes GJ Pifarre R Fisher RI Increased incidence of lymphoproliferative disorder after immunosuppression with the monoclonal antibody OKT3 in cardiac-transplant recipients N Engl J Med 1990 323 1723 28 2100991 10.1056/NEJM199012203232502

[B22] Constanzo-Nordin MR O'Sullivan EJ Hubbell EA Zucker MJ Pifarre R McManus BM Winters GL Scanlon PJ Robinson JA Long-term follow-up of heart transplant recipients treated with murine antihuman mature T-cell monoclonal antibody (OKT3): the Loyola experience J Heart Transplant 1989 8 288 95 2504895

[B23] Bourge RC Kirklin JK McGriffin DC Total lymphoid irradiation after cardiac transplantation: is there a risk of late leukaemia [abstract] J Heart Lung Transpl Program Issue 1998

[B24] Kim KW Hwang M Moretti L Jaboin JJ Cha YI Lu B Autophagy upregulation by inhibitors of caspase-3 and mTOR enhances radiotherapy in a mouse model of lung cancer Autophagy 2008 5 659 68 10.4161/auto.6058 PMC3073356 18424912

[B25] Murphy JD Spalding AC Somnay YR Markwart S Ray ME Hamstra DA Inhibition of mTOR radiosensitizes soft tissue sarcoma and tumor vasculature Clin Cancer Res 2009 15 589 96 10.1158/1078-0432.CCR-08-1019 19147764

